# A 4D Printable Shape Memory Vitrimer with Repairability and Recyclability through Network Architecture Tailoring from Commercial Poly(*ε*‐caprolactone)

**DOI:** 10.1002/advs.202103682

**Published:** 2021-10-29

**Authors:** Jungho Joe, Jeehae Shin, Yong‐Seok Choi, Jae Hyuk Hwang, Sang Hwa Kim, Jiseok Han, Bumsoo Park, Woohwa Lee, Sungmin Park, Yong Seok Kim, Dong‐Gyun Kim

**Affiliations:** ^1^ Advanced Materials Division Korea Research Institute of Chemical Technology 141 Gajeong‐ro, Yuseong‐gu Daejeon 34114 Republic of Korea; ^2^ Composite Materials Application Research Center Korea Institute of Science and Technology 92 Chudong‐ro, Bongdong‐eup Wanju‐gun Jeonbuk 55324 Republic of Korea; ^3^ School of Chemical and Biological Engineering and Institute of Chemical Processes Seoul National University 599 Gwanak‐ro, Gwanak‐gu Seoul 08826 Republic of Korea; ^4^ Department of Chemical Engineering and Applied Chemistry Chungnam National University 99 Daehak‐ro, Yuseong‐gu Daejeon 34134 Republic of Korea; ^5^ Department of Polymer Engineering Chungnam National University 99 Daehak‐ro, Yuseong‐gu Daejeon 34134 Republic of Korea; ^6^ Advanced Materials and Chemical Engineering KRICT School University of Science and Technology 217 Gajeong‐ro, Yuseong‐gu Daejeon 34114 Republic of Korea

**Keywords:** 4D printing, fused deposition modeling, poly(*ε*‐caprolactone), polymer recycling, vitrimer

## Abstract

Vitrimers have shown advantages over conventional thermosets via capabilities of dynamic network rearrangement to endow repairability as well as recyclability. Based on such characteristics, vitrimers have been studied and have shown promises as a 3D printing ink material that can be recycled with the purpose of waste reduction. However, despite the brilliant approaches, there still remain limitations regarding requirement of new reagents for recycling the materials or reprintability issues. Here, a new class of a 4D printable vitrimer that is translated from a commercial poly(*ε*‐caprolactone) (PCL) resin is reported to exhibit self‐healability, weldability, reprocessability, as well as reprintability. Thus, formed 3D‐printed vitrimer products show superior heat resistance in comparison to commercial PCL prints, and can be repeatedly reprocessed or reprinted via filament extrusion and a handheld fused deposition modeling (FDM)‐based 3D printing method. Furthermore, incorporation of semicrystalline PCL renders capabilities of shape memory for 4D printing applications, and as far as it is known, such demonstration of FDM 3D‐printed shape memory vitrimers has not been realized yet. It is envisioned that this work can fuel advancement in 4D printing industries by suggesting a new material candidate with all‐rounded capabilities with minimized environmental challenges.

## Graphical abstract

A widely utilized commercial 3D printing resin poly(*ε*‐caprolactone) (PCL) is translated into a PCL‐based vitrimer via end‐group crosslinking and transesterification‐based vitrimerization. The formed vitrimer enables dynamic network rearrangement for self‐healing, welding, reprocessing, and viscoelastic flowing. Furthermore, endowed mechanical strength and shape memory effects in comparison to the commercial PCL resin open up a new era for multifunctional fused deposition modeling (FDM)‐based 4D printing.

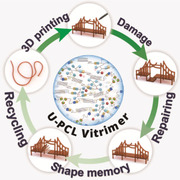

## 1 Introduction

Researches on 4D printing technologies have been extensively carried out for a wide range of applications including nanodevice manufacturing, soft robotics, and biomedical engineering.^[^ ^1^, ^2^, ^3^, ^4^, ^5^, ^6^ ^]^ 4D printing technology offers an extra feature of dynamic structural changes as a function of time when the objects are stimulated by an external cue.^[^ ^7^, ^8^, ^9^, ^10^, ^11^ ^]^ For example, a simple 2D‐to‐3D structural transition can be made^[^ ^12^, ^13^, ^14^ ^]^ or the configuration of a 3D‐printed product can be directly transformed,^[^ ^15^, ^16^ ^]^ further allowing us to design and control movements. Such shape‐shifting ability generally arises from reversible thermomechanical characteristics of shape memory polymers (SMPs).^[^ ^17^, ^18^, ^19^ ^]^ SMPs refer to a range of elastic polymeric networks that are either chemically crosslinked (e.g., thermosets)^[^ ^20^ ^]^ or physically entangled (e.g., block copolymers).^[^ ^21^ ^]^ In most of the cases of chemically networked SMPs, external stress at above the shape memory transition temperature (*T*_trans_, i.e., glass transition temperature (*T*_g_) or melting temperature (*T*_m_)) leads to macroscopic deformation of the permanent shape with a lowered entropy state, which can be subsequently fixed by freezing the polymer chains below the *T*_trans_. Thus yielded temporary shape can snap back to the original permanent shape at above the *T*_trans_, through allowing the chains to return to their highest entropy state.^[^ ^15^, ^22^, ^23^ ^]^ Recent studies presented thermally responsive shape memory behaviors of various materials for 4D printing applications. Fang et al. demonstrated configuration transitions from a 2D film to a 3D module, through selective photocuring and swelling.^[^ ^12^ ^]^ Since the constituting material contained dynamic hindered urea linkages and semicrystalline poly(*ε*‐caprolactone) (PCL) chains, they showed a seamless assembly of the created 3D modules via dynamic bond exchange reactions, as well as shape memory behaviors by thermomechanical stimulation. In another example, Ge et al. controlled the material components to precisely tune the resulting *T*_g_, and utilized microstereolithography (SLA) to build multimaterial 3D structures.^[^ ^15^ ^]^ The resulting products exhibited thermally induced shape memory behaviors and further presented actuation for soft‐robotic applications.

Likewise, with the sharp increase of interests and development in 3D and 4D printing fields, there also has been a rising awareness to seek for reprocessing methods to regenerate the products that are expired. A breakthrough to impart such abilities is the introduction of a vitrimer, a polymeric network connected by associative dynamic covalent bonds.^[^ ^24^, ^25^, ^26^, ^27^ ^]^ Vitrimers show unique physicochemical characteristics in which the network maintains a fixed crosslinking density while dynamic covalent bonds can actively exchange at above the topology‐freezing transition temperature (*T*_v_).^[^ ^28^ ^]^ Leibler's group first introduced dynamic network rearrangement via transesterification reactions in 2011,^[^ ^26^ ^]^ and ever since, versatile material compositions and thermomechanical properties of the resulting vitrimers have been investigated to realize material self‐healing, welding, and reprocessing.^[^ ^29^, ^30^, ^31^, ^32^ ^]^ Previous studies have successfully applied vitrimers to 3D printing in order to alleviate growing environmental concerns. For instance, Shi et al. partially cured epoxy vitrimer network to form highly viscous and printable ink.^[^ ^32^ ^]^ The printed product via direct ink writing (DIW) method was further cured at a high temperature to fully crosslink the network and enhance mechanical strength. To recycle, an excess amount of ethylene glycol was added to depolymerize the formed structure back to the precursors via transesterification reactions for the next round of printing. In another example, Zhang et al. reported a two‐step process to produce reprocessable 3D thermosets.^[^ ^30^ ^]^ Initial 3D thermosets were printed through a UV curing‐based SLA method, and then they were heated to induce additional transesterification‐based network rearrangements, rendering both material stiffness and recyclability. The reported studies thus far offer promises for the potential use of vitrimers in reprocessing 3D‐printed products; however, there still remain limitations to access reprintability without requiring new reagents that consequently may alter material compositions and the following future printing quality. We also note that most current research attempts are yet restricted to formulating reprocessable 3D printing ink, and less attention is given to exploring 4D printable material equipped with additional functions that a vitrimer possesses as well as facile fused deposition modeling (FDM)‐based (re)printability.

**Scheme 1:**
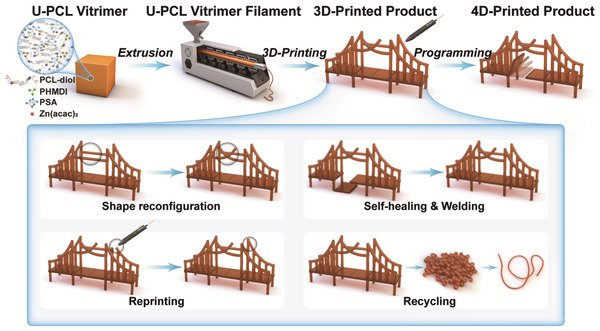
Schematic representation of a 4D printing process of a U‐PCL vitrimer via filament extrusion and handheld FDM‐based 3D printing, and its multiple functions including shape reconfiguration, self‐healing, repair by welding and reprinting, and recycling.

In this paper, we expand the concept of a reprocessable 3D printing vitrimer ink and report a new class of a 4D printable PCL‐based vitrimer. PCL is known for one of the broadly available commercial resins in FDM‐based 3D printing;^[^ ^33^, ^34^ ^]^ hence, developing the commercial PCL into a vitrimer earns merits in using various types of heat‐activated systems. To synthesize PCL‐based vitrimer, PCL with hydroxyl end groups (PCL‐diol) is first covalently crosslinked with isocyanate groups of poly(hexamethylene diisocyanate) (PHMDI) with the aid of a zinc acetylacetonate (Zn(acac)_2_) catalyst; the generated urethane‐networked PCL (U‐PCL) compensates the low mechanical property of thermoplastic linear PCL and concurrently endows shape memory capabilities. The network is then adapted to a U‐PCL‐based vitrimer (U‐PCL vitrimer) by introducing a low‐molecular‐weight poly(styrene‐*co*‐allyl alcohol) (PSA) with the average number of six hydroxyl groups per chain as a dynamic crosslinker to react with ester functionalities of PCL via transesterification at a high temperature. The use of PSA is found to improve the network connectivity by preventing excessive fragmentation during the dynamic network rearrangement in comparison to three hydroxyl group‐containing small molecules (e.g., glycerol).^[^ ^37^ ^]^ The network components are finely tuned to maximize transesterification efficiency while retaining PCL crystallinity. Resulting U‐PCL vitrimers are shown to gain self‐healability at a fast rate, facile weldability, repeated reprocessability, and consecutive transitions between elasticity‐based shape memory and plasticity‐based reconfiguration. We also find that these characteristics are consistent in various fabrication forms including films, extruded filaments, and complex 3D structures constructed by a portable 3D pen. Since our printing system does not involve photopolymerization and does solely depend on viscoelastic flowability dictated by the dynamic bond exchange reactions of the U‐PCL vitrimer, it further allows for reprinting without extra additives and cross‐fabricable recycling (e.g., from films to 3D printing) (**Scheme** **1**). To the best of our knowledge, the vitrimer application to FDM‐based 4D printing is demonstrated for the first time. We envision our 4D printable U‐PCL vitrimer with all‐rounded capabilities, spanning from shape reconfiguration, self‐healing, repair by welding and reprinting, to recycling, will suggest guidance to advancement in the future 4D printing researches and industries.

## 2 Results and Discussion

### 2.1 Synthesis and Characterization of U‐PCL Vitrimer

**Figure 1:**
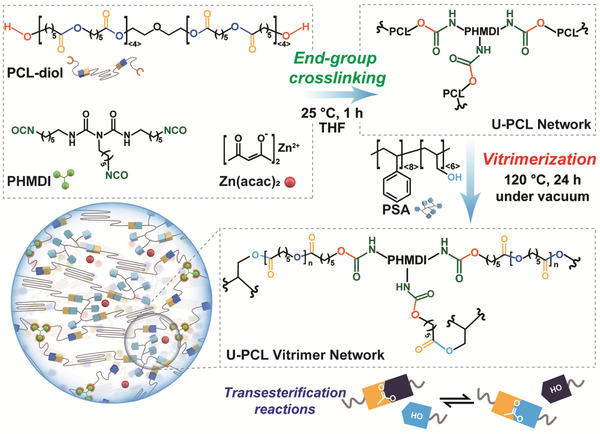
Synthesis of a U*_a_*‐PCL‐based vitrimer via stepwise end‐group crosslinking and vitrimerization.

The overall U‐PCL vitrimer synthesis process is presented in **Figure** **1**. First, we converted a conventional 3D printing resin, PCL, to a U‐PCL network. PHMDI used in the study contains the average number of three isocyanate groups per molecule that are able to crosslink with hydroxyl‐terminated PCL and yield urethane linkages.^[^ ^35^ ^]^ Since PCL is highly fluidic at above *T*_m_, the initial networking process provides reliable mechanical strength to prevent material melting‐induced flowing at an elevated temperature while maintaining the ester groups in the PCL main chain for transesterification reactions. We varied the molar ratios of PHMDI to PCL‐diols across the series as listed in Table S1 in the Supporting Information. Here, we designate the tested molar ratio of [NCO]/[OH] as subscripted “*a*,” and present the respective network as U*_a_*‐PCL. The organozinc catalyst, Zn(acac)_2_, mediates the urethane network formation,^[^ ^36^ ^]^ thus the initial mol% of Zn^2+^ was fixed to 2 with respect to the total number of Zn(acac)_2_ molecules and the ester groups from PCL. After being stirred at 25 °C in a tetrahydrofuran (THF) solvent for 1 h and dried under vacuum for 2 h, the reaction mixtures were transformed to a U*_a_*‐PCL network and the process was monitored by Fourier transform infrared (FT‐IR) spectroscopy. When “*a*” was 1.7 or below, the peak from PHMDI, corresponding to NCO stretching vibration at 2270 cm^−1^, disappeared, confirming full participation of PHMDI into crosslinking (Figure S1, Supporting Information). We also hypothesized that a higher level of PHMDI to form robust urethane linkages can produce the network backbone with increased mechanical strength as a vitrimer precursor. To test the mechanical strength, we fabricated the series into films and folded them to identify any breakage (Figure S2, Supporting Information). As we speculated, when “*a*” was 1.3 or below, the films were broken and we attributed this to the possibility of inadequate network formation with the remained unreacted PCL‐diol. Indeed, when it came to *a* = 1.5 or 1.7, the generated films withstood the stress from being folded. Furthermore, after the network formation, PCL crystallinity was found to be maintained by differential scanning calorimetry (DSC) analyses across the series (Table S2, Supporting Information). Based on the observations, to maximize the network crosslinking without residual isocyanate groups while maintaining crystallinity, we selected U_1.7_‐PCL as the vitrimer precursor.

**Figure 2:**
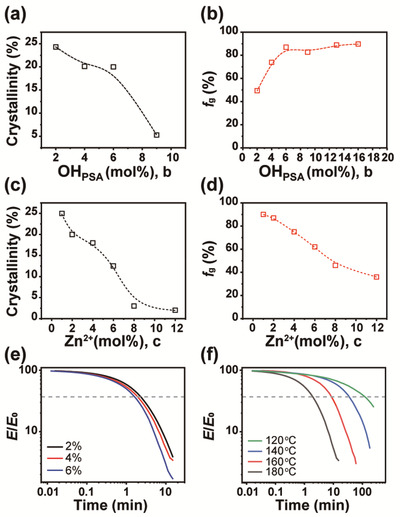
Effects of PSA and Zn(acac)_2_ contents on the physical properties and stress relaxation behaviors of U‐PCL vitrimers. Plots of a) crystallinity versus PSA content and b) gel fraction versus PSA content of U_1.7_‐PCL‐PSA*_b_* vitrimers. Plots of c) crystallinity versus Zn(acac)_2_ content and d) gel fraction versus Zn(acac)_2_ content of U_1.7_‐PCL‐PSA_6_‐Zn*_c_* vitrimers. e) Iso‐strain stress relaxation curves of U_1.7_‐PCL‐PSA_6_‐Zn*_c_* (*c* = 2%, 4%, and 6%) vitrimers at 180 °C. f) Iso‐strain stress relaxation curves of U_1.7_‐PCL‐PSA_6_‐Zn_4_ vitrimer at different temperatures. The dashed gray‐colored line indicates *σ*/*σ*_0_ = *e* ^−1^ (≈37% of the initial stress).

The second network was constructed through vitrimerization facilitated by transesterification reactions between ester and hydroxyl functionalities. Previously, small molecules with three hydroxyl groups, such as glycerol, have been utilized in vitrimerization of ester‐containing high temperature polymers (e.g., poly(butylene terephthalate));^[^ ^37^ ^]^ however, when applied for the U‐PCL vitrimer preparation, we observed loss of crystallinity as well as decreased gel fraction (*f*_g_) value of the product, possibly due to excessive fragmentation of the network structure. To overcome the addressed issue, we adopted PSA with the average number of six hydroxyl groups per chain to react with ester groups within the PCL chains for vitrimerization. We examined the effect of varied PSA molar concentrations, as listed in Table S3 in the Supporting Information, on the resulting physical properties of the created U_1.7_‐PCL‐PSA*_b_*. Here, subscripted “*b*” represents the molar percentage of hydroxyl groups of PSA per the total moles of ester groups from PCL and hydroxyl groups from PSA. The series of U_1.7_‐PCL‐PSA*_b_* vitrimers were prepared through transterification reactions between PSA and the U‐PCL network at 120 °C for 24 h under vacuum, following the addition of dilute PSA in THF into the reaction solution of U_1.7_‐PCL. We first tracked changes in the consequent *T*_m_ by DSC analyses across the series and distinctive *T*_m_ values were detected only when “*b*” values ranged from 2 to 6 mol% (Figure S3a and Table S4, Supporting Information). With the increase in the involved PSA content, the respective *T*_m_ shifted to a lower temperature and the sharpness of the peak also decreased. We reason this to more fragmented and constrained PCL chains due to the growing crosslinking density, hence hindering PCL crystallization in addition to the decrease of PCL content itself in the vitrimers. Decreasing crystallinity of the vitrimers was also in agreement with the decreasing trend of *T*_m_ (**Figure** **2**a). To examine possible crystalline structure changes of a range of PSA concentrations, a wide angle X‐ray diffraction (WAXD) analysis was conducted. As shown in Figure S4a in the Supporting Information, characteristic diffraction peaks of the PCL crystal structure were observed without position shifts.^[^ ^38^ ^]^ The crystallinity evaluated from 1D WAXD profiles also showed a behavior similar to the result from DSC, where the crystallinity decreases as the incorporated hydroxyl content increases (Figure S4c, Supporting Information).^[^ ^38^ ^]^ We next obtained the resulting gel fraction values to evaluate the final vitrimer network integrity led by PSA variations. As indicated in Figure 2b, higher PSA contents augmented the crosslinking reaction and the corresponding gel fraction. Since too high of a concentration was in turn found to disfavor PCL crystallization, we chose 6 mol% of PSA to acquire sufficient transesterification‐induced network integrity while preserving PCL crystallinity.

After completing the general network design, we precisely controlled transesterification reaction rates. We considered two factors that mediate the reactions: temperatures and organozinc catalyst concentrations. In the first step, we modulated the level of Zn(acac)_2_ catalyst that governs both end‐group crosslinking and transesterification processes. Increasing the Zn(acac)_2_ content in the vitrimer was expected to accelerate transesterification rates that further affect the total time paid for self‐repair and reprocessing.^[^ ^39^ ^]^ Although it was of crucial importance to shorten the transesterification reaction time, it was also desirable to obviate the loss of crystallization caused by the excess amount of the incorporated catalyst. We prepared a set of U_1.7_‐PCL‐PSA_6_ consisting of the varied Zn^2+^ levels ranging from 1 to 12 mol% as listed in Table S5 in the Supporting Information, and explored *T*_m_ changes via DSC analyses (Figure S3b, Supporting Information). Except for 12 mol%, all candidates showed distinctive melting points; but the degree of crystallinity declined drastically when 6 mol% or higher levels were applied, presumably due to the excessive catalyst content interfering crystallization (Figure 2c). WAXS analyses further supported the results by revealing the absence of characteristic diffraction peak shifting of PCL as well as the similar decreasing tendency of the evaluated crystallinity (Figure S4b,d, Supporting Information). Moreover, while the increase of PSA promoted gelation via denser network formation (Figure 2b), the increase in the Zn^2+^ content exhibited a reversed trend as depicted in Figure 2d, possibly incurred by the excessive branching reactions. To examine the dynamic network rearrangement capabilities for both repairability and reprocessability of the materials, we prepared the selected 2, 4, and 6 mol% of Zn^2+^‐containing vitrimer films via simple hot pressing at 170 °C under 10 MPa for 30 min, and conducted iso‐strain stress relaxation experiments under the fixed temperature of 180 °C. As anticipated, faster stress‐relaxation behaviors in the order of 6, 4, and 2 mol% were monitored and this confirmed the critical effects of the catalyst concentration on transesterification kinetics (Figure 2e). Although 6 mol% showed the best network rearrangement capabilities among the three samples, considering the consequent low crystallinity and gel fraction, we selected 4 mol% of Zn^2+^. In the second step, we studied the temperature‐dependent transesterification rates with a fixed 4 mol% of Zn^2+^. Figure 2f shows that the applied temperature decrease also had a strong impact in decelerating the transesterification reactions. The result suggests that at least 140 °C should be applied to allow adequately fast, complete stress relaxation behaviors and therefore to achieve meaningful effects we expect from the transesterification reactions.

### 2.2 Self‐Healability, Weldability, and Reprocessability of U‐PCL Vitrimer

**Figure 3:**
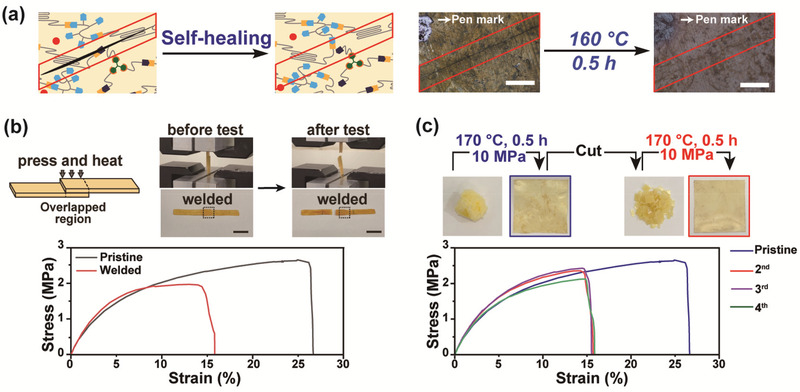
Self‐healing, welding, and reprocessing of U‐PCL vitrimers. a) Optical microscopy images of a scratched U_1.7_‐PCL‐PSA_6_‐Zn_4_ film before (0 min) and after (30 min) heat treatment at 160 °C (scale bar: 500 µm). b) Schematic illustration of thermal welding and photographs of before and after the lap‐shear test; stress–strain curves of pristine and thermally welded U_1.7_‐PCL‐PSA_6_‐Zn_4_ films (scale bar: 10 mm). c) Photographs of reprocessed vitrimer films via hot pressing and the respective stress–strain curves of reprocessed U_1.7_‐PCL‐PSA_6_‐Zn_4_ films (blue squared film: first reprocessed pristine film; red squared film: second reprocessed film).

One of the anticipated effects we benefit from dynamic network rearrangement is the temperature‐induced self‐repairing. To demonstrate such function, we prepared a scratched U_1.7_‐PCL‐PSA_6_‐Zn_4_ vitrimer film and incubated it at 160 °C for 30 min. According to **Figure** **3**a, when the defected film was heat‐treated, the scratched gap was filled up via polymer chain mobility and active transesterification reactions. The surface depth profiling shown in Figure S6 in the Supporting Information, also revealed that the scratch with a depth of ≈5 µm was successfully recovered to its original state. We further attempted to optically compare the healing speed of the given scratched films containing different catalyst contents. In agreement with the previous stress relaxation data, the vitrimer film with 2 mol% Zn^2+^ resulted in incomplete surface recovery while the vitrimer film with 4 mol% Zn^2+^ sufficiently healed the defected surfaces when exposed at 160 °C for only 1 min (Figure S7, Supporting Information). In addition, we modulated both temperature and time parameters to investigate healing speeds of the scratched vitrimer films with 2 and 4 mol% catalyst by gradually heating the materials from 30 to 160 °C at a heating rate of 10 °C min^−1^ (Figure S8, Supporting Information). Both films initially showed little changes at 30 °C but the latter case started to show notably faster repairing when the temperature reached 80 °C and mostly progressed recovery at 160 °C. The former case also exhibited healing capabilities at 80 °C; however, due to much slower progression, only a small portion was recovered at 160 °C, reiterating the importance of the applied catalyst concentrations in the resulting transesterification efficiency.

In addition to self‐repair abilities, the vitrimer films also showed welding capabilities at the interface, and to prove it, we performed lap‐shear tests. As schematically illustrated in Figure 3b, two vitrimer films were overlapped, pressed, and then incubated at 160 °C for 2 h to induce interfacial transesterification reactions. Next, the tensile property of the welded film was tested by universal testing machine (UTM). The obtained stress–strain curve of the welded products showed a similar deformation trend but with much smaller strain at break comparing to the pristine film's (Figure 3b; Table S7, Supporting Information). This could be attributed to the increased thickness, and therefore increased stiffness, of the overlapped area that could restrict the film extension. After the testing, we identified the film had a breakage outside the welded region and the welded area remained firmly intact (Figure 3b). The presented welding feature exists only in vitrimers and is clearly distinguished from other conventional recyclable thermoplastics since thermoplastics are typically deformed or melted by heat treatment. Vitrimers, on the other hand, can be welded locally, without collapsing the overall structure, by the interfacial dynamic network rearrangement, providing convenient options to the users how to repair the products.

Reprocessability is another practical way of proving the network rearrangement capabilities. Since our vitrimer is aimed for complete recycling, which is independent of involving a depolymerization process but relying on the transesterification reactions, reprocessing capabilities were investigated via a simple hot pressing method. First, we prepared a U_1.7_‐PCL‐PSA_6_‐Zn_4_ vitrimer in a bulk state through the synthetic procedure, and pressed it for film fabrication at 170 °C under 10 MPa for 30 min. The bulk vitrimer underwent the dynamic network rearrangement and transformed into the first reprocessed pristine film. We repeated the same fabrication cycles, pelletization and hot pressing, to demonstrate reprocessability, and indeed the film was successfully regenerated (Figure 3c). When we analyzed stress–strain curves of the reprocessed samples by UTM, we noticed that the stress–strain curves showed the decreasing maximum strain of the reprocessed samples comparing to the first reprocessed film from the bulk vitrimer (Figure 3c; Table S8, Supporting Information). We carefully reasoned this to the highly entangled polymeric chains of the initial film that was directly fabricated from the as‐synthesized vitrimer in the bulk state. The entanglement might have partly resolved through fabrication processes at the high temperature and the following repelletization, resulting in the strain reduction. Our presumption can be further supported by the consistent stain values of the subsequently reprocessed films. Since we were able to repeatedly reprocess the films, it was convincing that the transesterification reactions were firmly taking place, and furthermore, DSC analyses revealed that the reprocessed films still maintained *T*_m_ and crystallinity within a reasonable range, which is required to endow shape memory effects for 4D printing (Figure S9, Supporting Information).

### 2.3 Thermadapt Shape Memory Behavior of U‐PCL Vitrimer

**Figure 4:**
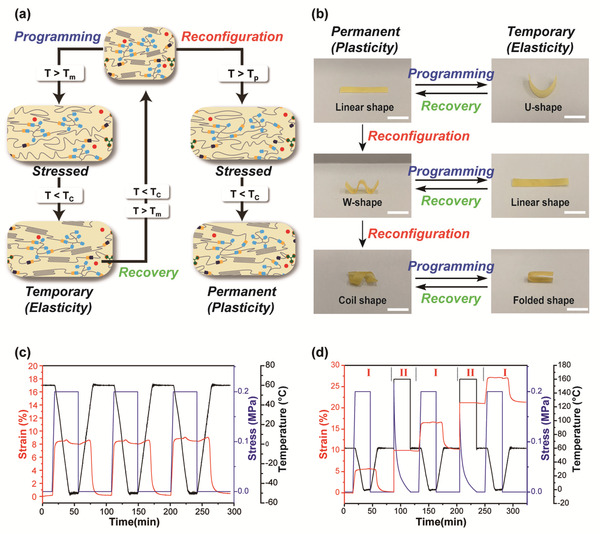
Thermadapt shape memory behavior of U‐PCL vitrimer. a) Schematic illustration of the thermally distinct shape‐memory and reconfiguration. b) Complex shape manipulation of U_1.7_‐PCL‐PSA_6_‐Zn_4_ vitrimer via plasticity‐based cumulative shape reconfiguration and elasticity‐based shape memory behaviors (scale bar: 10 mm). c) Quantitative shape memory cycles of U_1.7_‐PCL‐PSA_6_‐Zn_4_ vitrimer. d) Consecutive elasticity and plasticity cycles of U_1.7_‐PCL‐PSA_6_‐Zn_4_ vitrimer (regions marked with I and II represent elasticity and plasticity cycles, respectively).

After validation of repairability, weldability, and reprocessability of the synthesized U‐PCL vitrimer films, we studied shape‐memory capabilities. To apply our vitrimer to 4D printing, heat‐activated SMP‐based actuation is required. In this system, such shape memory behavior is manipulated by the crystalline phase of PCL domains and thus we carefully preserved *T*_m_ and crystallinity during the vitrimer synthesis. Shape memory capabilities are endowed through an elastic thermomechanical programming process. Deformation of the vitrimer in its pristine permanent shape at above *T*_m_, followed by recrystallization below *T*_c_, yields a temporary shape. When the temporary shape is again subjected to heat above *T*_m_, it is thermodynamically driven to return to the original shape via exhibiting elasticity. In addition to the elasticity‐based shape memory behavior, the vitrimer can also undergo plasticity‐based shape reconfiguration when the temperature is applied at above the plasticity temperature (*T*_p_) where the network rearrangement is induced by dynamic transesterification reactions. The *T*_p_ can be considered as a dynamic experimental temperature at which plasticity is induced at a significant rate within the experimental time scale, rather than a static temperature.^[^ ^40^ ^]^ The SMPs, capable of thermally distinct plasticity‐based shape reconfiguration as well as elasticity‐based shape memory behaviors, are known as thermadapt SMPs.^[^ ^41^ ^]^ The thermadapt SMP characteristics, including shape memory and reconfiguration behaviors, are schematically summarized and illustrated in **Figure** **4**a.

A series of permanent as well as programmed temporary shape shifting was demonstrated in Figure 4b. Initially the U_1.7_‐PCL‐PSA_6_‐Zn_4_ vitrimer in its permanent configuration was deformed to a desired shape at 60 °C, followed by cooling to −20 °C for fixing the temporary shape. Shape memory effects were then triggered by subjecting the deformed vitrimer to 60 °C to retain its original morphology. Shape reconfiguration was carried out at 160 °C for 10 min and subsequently cooled down to fix the structure. We showed three differently designed permanent configurations and the according shape fixing/recovery of each state, which substantiated both capabilities of repeated plasticity and elasticity of the vitrimer (Movie S1, Supporting Information). To further corroborate, we conducted dynamic mechanical analysis (DMA) experiments to examine repetitive shape memory behaviors and sequential shape memory‐shape reconfiguration cycles. In agreement with the previous observations, Figure 4c,d clearly shows stable shape memory cycles and a series of elasticity‐based shape memory and plasticity‐based cumulative reconfiguration cycles, respectively.

### 2.4 4D Printing of U‐PCL Vitrimer

**Figure 5:**
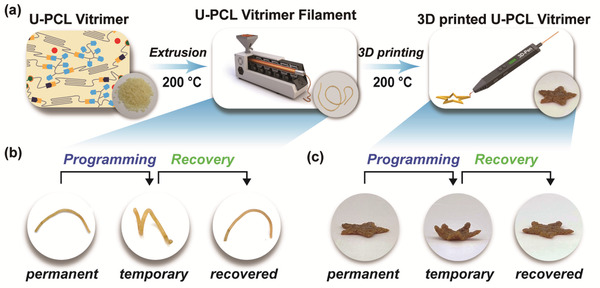
Filament extrusion and 3D/4D printing of U‐PCL vitrimers. a) 3D printing process using a 3D pen with U‐PCL vitrimer filament. Shape memory effects of b) extruded filament and c) star‐shape printed structure from U_1.7_‐PCL‐PSA_6_‐Zn_4_.

The next objective of this work was to examine viscoelastic flow behavior of the vitrimers and apply them to handheld FDM‐based 4D printing applications. The excellent dynamic network rearrangement capabilities exhibited by the vitrimers showed promises in continuous processing (Figure 2f). The presence of a branched phase, which is not fully crosslinked and corresponding to a soluble fraction (≈25%) in U_1.7_‐PCL‐PSA_6_‐Zn_4_ vitrimer, was also expected to act as a lubricant.^[^ ^42^ ^]^ Prior to actual heat‐activated extrusion for 3D printing, we first tested feasibility of continuous processing of the vitrimers by conducting melt‐flow‐index experiments at 204, 207, and 210 °C. The amount of the extruded materials for 10 min under the given temperature was quantified to be around 18.5, 24.5, and 31.5 g, respectively (Figure S10, Supporting Information). Comparing to the previously reported literature, these values were found to be adequately rapid for extrusion‐based processing systems.^[^ ^43^, ^44^ ^]^ Based on the melt‐flow index and thermogravimetric analysis (TGA) data, we considered 200 °C to be suited for further extrusion experiments to prevent any possible decomposition (Figure S5, Supporting Information). Before generating the vitrimer filaments, we also investigated its rheological property by a frequency sweep experiment with 1% strain at 200 °C. As shown in Figure S11 in the Supporting Information, shear rate dependent complex viscosity (η*) was observed in our vitrimer. The decreasing of η* at a high shear rate helps smooth extrusion at the nozzle during printing, while the recovered high η* after extrusion (at zero‐shear) allows to retain the printed shape. Thus the shear rate dependency of our vitrimer confirmed feasibility of FDM‐based printing.^[^ ^6^, ^45^ ^]^ Next, as illustrated in **Figure** **5**, we generated vitrimer filaments by a mono‐screw‐equipped extruder and the extruded filament was found to show the same shape memory effects as the films did (Figure 5b). We then applied the filaments to a portable 3D pen to create artwork (Movie S2, Supporting Information). The 3D printed, star‐shaped product was also able for shape programming and recovery, leading to 4D printing realization (Figure 5c).

**Figure 6:**
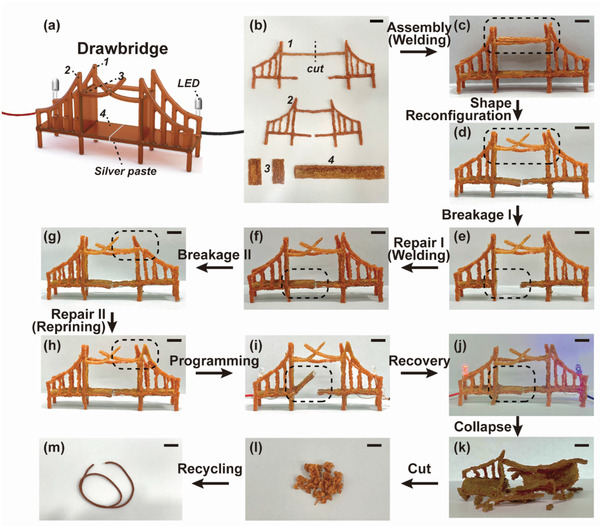
Demonstration of multifunctionality of 3D‐printed architecture from U‐PCL vitrimer. a) Schematic representation of drawbridge structure prepared via handheld FDM‐based 3D printing and welding. b) Assembly components of the drawbridge. c) Assembled drawbridge through welding. d) Shape reconfiguration. e) Broken bridge. f) Repaired bridge through welding. g) Broken bridge. h) Repaired bridge through reprinting. i) Shape programming. j) Shape recovery. The site for each shape manipulation is indicated as a dotted circle. k) Collapsed vitrimer bridge. l) Cut bridge pieces for recycling. m) Reprocessed vitrimer filament for reprinting (scale bar: 10 mm).

We thus far proved each function of the synthesized vitrimer sequentially. As the final demonstration, we built a drawbridge architecture with a 3D pen and examined its dimensional stability at elevated temperatures in comparison to the commercial PCL resin as well as the equipped functions including healing, shape memory, and recycling (**Figure** **6**a). First, two identical bridges were created where the first type consists of commercial thermoplastic PCL and the latter consists of our vitrimer. Commercial PCL filaments have been widely utilized in FDM‐based 3D printing industries; however, their low mechanical stability at high temperatures, originating from the low *T*_m_ (≈60 °C), often discourages the use. To show superior mechanical stability of the vitrimer, both types of the bridges were exposed to 80 °C for 5 min (Figure S12, Supporting Information). As anticipated, the bridge made out of commercial thermoplastic PCL collapsed after heat treatment, while the vitrimer bridge held the structure in place suggesting strong heat resistance rendered by the tightly crosslinked network (Movie S3, Supporting Information).

We then verified the accompanied functions of the vitrimer bridge. The bridge was built by assembling each 3D‐printed component via welding (Figure 6b,c). Thus constructed bridge was able to display shape reconfiguration and repairing via welding and/or reprinting due to the active transesterification reactions at the interfaces (Figure 6d–h). To further demonstrate 4D printing, we designed the bridge with the disconnected center, where two separate panels can be ramped or docked depending on the permanent/temporary shape designation. Here, the docked/connected panels were set as a permanent shape and the ramped/separated panels were fixed as a temporary shape. We additionally applied conductive silver paste under the bridge and placed small light bulbs at both ends of the bridge (Figure 6a). When the bridge with the programmed panels (i.e., separated shape) was subjected to heat, the ramped panel moved down to its original state (i.e., connected shape) (Movie S4, Supporting Information). After the shape recovery, both light bulbs turned on as the evidence of the panel connection, which is illustrated in Figure 6i,j. This suggests any counterparts in the complex structure can be designed for actuation, which can provide unlimited potential in the material design. Last, to demonstrate full recycling, we utilized the damaged and collapsed vitrimer bridge. Through the same pelletization and extrusion, we were able to recycle the printed waste to a new filament for 3D printing (Figure 6k–m). This process can be repeatedly performed to realize reusable, thus eco‐friendly 4D printing resins.

## 3 Conclusion

In this work, we utilized a 3D printing commercial resin, PCL, and simply generated a PCL‐based vitrimer network by introducing crosslinkers and catalysts. Diol‐containing PCL chains were first converted to a urethane‐networked PCL by isocynate‐containing PHMDI crosslinkers in the presence of Zn(acac)_2_ catalysts to impart mechanical strength. Then PSA with hydroxyl functionalities were added to induce vitrimerization through transesterification reactions. The resulting vitrimer was found to show superior mechanical properties and exhibit self‐healing, welding, and recycling capabilities. Due to the excellent viscoelastic flow behavior of the vitrimer originating from fast stress relaxation at a high temperature, we were able to extrude filaments and generate 3D printed structures by a portable 3D pen. Furthermore, taking advantages of PCL crystallinity, we endowed shape memory effects and successfully developed 4D printable materials with all‐rounded benefits. We believe that the developed system can be applied to a universal FDM‐based 3D printer, thus we are currently working on scaling‐up the vitrimer filament production and creating complex structured products by a 3D printer. Taking one step further, we expect considerable potential of our vitrimer as a composite material in combination with versatile organic or inorganic fillers to introduce extra desirable functions.

## 4 Experimental Section

### Materials

PCL‐diol (*M_n_*_,_ = 2000 g mol^−1^), commercial PCL filament (1.75 mm × 5 m, AB Link), PHMDI (viscosity = 1300–2200 cP@25 °C, NCO content = 22.6–23.7%), zinc acetylacetonate hydrate (Zn(acac)_2_), and PSA (*M_n_* = 1200 g mol^−1^, allyl alcohol 40 mol%) were purchased from Sigma‐Aldrich. THF (stabilized, >99.9%) was purchased from Samchun Chemicals. All other reagents and solvents were in commercial grade and used as received without further purification.

### Synthesis of U‐PCL Network

Urethane‐based poly(*ε*‐caprolactone) networks were designated as U*_a_*‐PCLs, where “*a*” indicated the molar ratio of [NCO]/[OH]. The chemical components of the following set of U*_a_*‐PCL are listed in Table S1 in the Supporting Information. As a general procedure for U_1.7_‐PCL synthesis, PCL (2.500 g, 1.250 mmol), Zn(acac)_2_ (0.118 g, 0.448 mmol), and THF (5 mL) were added into a 20 mL scintillation vial. Then, diluted PHMDI (0.791 g, 1.453 mmol) in THF (2 mL) was slowly added to the mixture and stirred for 1 h at 25 °C to form the U‐PCL network. For analyses of the networks, THF in the final product was removed in a vacuum oven at 60 °C for 2 h.

### Synthesis of U‐PCL Vitrimer

U‐PCL‐based vitrimers were designated as U_1.7_‐PCL‐PSA*_b_*, where “*b*” indicated the molar percentage of hydroxyl groups of PSA per the total moles of ester groups from PCL and hydroxyl groups from PSA. Here, the molar ratio of [NCO]/[OH] for the initial U‐PCL network preparation was fixed at 1.7. Note that initially, Zn(acac)_2_ molar feed percentage was fixed to 2 with respect to the total molar content of Zn(acac)_2_ molecules and ester linkages of PCL. The chemical ingredients used to create a set of U_1.7_‐PCL‐PSA*_b_* are listed in Table S4 in the Supporting Information. In general, to synthesize U_1.7_‐PCL‐PSA_6_, PCL (2.500 g, 1.250 mmol), Zn(acac)_2_ (0.118 g, 0.448 mmol), and THF (5 mL) were added into a 20 mL scintillation vial. Subsequently diluted PHMDI (0.791 g, 1.453 mmol) in THF (2 mL) was slowly added into the mixture. After 1 h reaction, diluted PSA (0.260 g, 0.217 mmol) in THF (2 mL) was added into the mixture. The resulting mixture was then placed in a vacuum oven at 60 °C for 2 h to evaporate THF solvent. Last, the temperature was raised to 120 °C to induce transesterification for the next 24 h. In the case of U_1.7_‐PCL‐PSA_6_‐Zn*_c_*, hydroxyl content of PSA was fixed to 6 mol% and the catalyst concentrations were further modulated (Table S5, Supporting Information).

### Processing of U‐PCL Vitrimer

As‐synthesized vitrimers were applied to selective fabrication systems to yield films or filaments.

Fabrication of U‐PCL vitrimer film: U‐PCL vitrimer films were prepared by placing the polymers into a mold (e.g., 30 mm (*L*) × 30 mm (*W*) × 0.3 mm (*T*)), made of Kapton polyimide film and a stainless steel spacer mold, followed by pressing (10 MPa) at 170 °C for 30 min. For reprocessing, U‐PCL vitrimer films were cut into small pieces and repressed to the initial mold using the same procedure. All the films were additionally dried at 70 °C under vacuum for 12 h before analyses.

Filamentization and 3D printing of U‐PCL vitrimer: The prepared small pieces of U‐PCL vitrimer were added into a filament extruder (Filibot H303, Fordentech) and extruded at 200 °C to generate filamentized vitrimer. To construct 3D‐printed structure, a portable 3D pen (Sanago) operated at 200 °C was utilized.

### Studying Shape Memory Effects and Reconfiguration

To induce temporary shape deformation, U‐PCL vitrimer films or 3D‐printed materials were heated to 60^ ^°C, higher than their melting temperature (*T*_m_), for 5 min. Thus heated films or materials were deformed in the rubbery state and subsequently cooled to −20^ ^°C to fix the temporary shape via crystallization. The temporary shape was then heated to 60^ ^°C again to trigger the shape recovery to the permanent one. Reconfiguration was carried out by heating U‐PCL vitrimer films or 3D printed materials to 180 °C. Then heated films or materials were shape‐deformed at 180 °C for 10 min. The deformed films or materials were subsequently cooled to room temperature to obtain the reconfigured shapes. In order to firmly confirm the shape memory and recovery‐induced connectivity of a 3D‐constructed drawbridge, conductive silver paste (Elcoat P‐100) was applied. Small light bulbs were placed at each end of the bridge to test conductivity.

Quantitative shape memory behavior of U‐PCL vitrimer film (≈30 mm (*L*) × 5 mm (*W*) × 0.3 mm (*T*)) was also evaluated by TA Instruments DMA Q800 with an attached cryo accessory under the controlled force mode. The sample was first heated from 25 to 60 °C at a constant rate of 5 °C min^−1^ and maintained at 60 °C for 20 min. Next, the sample was stretched under a load of 0.2 MPa, followed by cooling to −50^ ^°C at a rate of 5 °C min^−1^ under the load (sample length = *ε*_load_) to fix the temporary shape. After unloading, the sample maintained the temporary shape with the length of *ε*_unload_. Shape recovery process was then triggered by heating the sample back to 60 °C at a rate of 5 °C min^−1^, which decreased the sample length to *ε*_rec_. Consecutive shape memory cycles were investigated by repeating the above shape memory cycle. Stress relaxation behaviors of U‐PCL vitrimers were analyzed by the same TA Instruments DMA Q800 under the stress relaxation mode. The samples were allowed to equilibrate at specified temperatures (120–180 °C) for 5 min, after which each sample was subjected to an instantaneous 10% strain. The stress relaxation was monitored, while maintaining the constant strain (10%), until the stress relaxation modulus had relaxed to at least 37% (1/*e*) of its initial value. Based on the thermally distinct plasticity and elasticity of U‐PCL vitrimers, combined elasticity/plasticity cycling test was performed by repeatedly switching the experimental modes (i.e., controlled stress and stress relaxation modes for elasticity and plasticity cycles, respectively) without unloading the sample after each cycle.

### Characterization

The thermal stability of U‐PCL vitrimers was investigated by TGA (TGA Q5000, TA Instruments) under a nitrogen atmosphere. The samples were heated to 800 °C at a heating rate of 10 °C min^−1^. A DSC (Q1000, TA Instruments) was run under a nitrogen atmosphere. Samples with a typical mass of 5–10 mg were encapsulated in sealed aluminum pans. They were first heated from −80 to 180 °C and then cooled down to −80 °C, which was followed by the second heating cycle at a constant rate of 10 °C min^−1^. Crystallinity was calculated with the obtained data following Equation (1)

(1) $$X_{c}\left(\%\right)=\left(\Delta H_{m}/\Delta H_{m}^{o}\right)\times 100\%$$

where Δ*H*_m_ is the second heating run melting endotherms, and Δ*H*°_m_ = 135 J g^−1^ (100% crystalline PCL).

WAXD measurement was performed using a Xenocs Xeuss 3.0 SAXS/WAXS equipment, and recorded on a 2D CCD detector (Dectris, Eiger2 R). The samples were located 0.20 m away from the detector, and 1.54 Å of wavelength X‐ray was used. 1D intensity profiles versus scattering vector *q* = (4*π*/*λ*)sin(*θ*/2) were obtained from collected 2D data, where *θ* is the scattering angle and *λ* is the wavelength of X‐ray. Crystallinity from WAXD (*X*_c‐WAXD_) was calculated using the following Equation (2)

(2) $$X_{c-\mathrm{WAXD}}\left(\%\right)=\left(I_{c}/I_{A}+I_{c}\right)$$

where *I*_c_ and *I*_A_ indicate integrated intensity at *q* range from 1.40 to 2.0 Å^−1^ for the crystalline peak and amorphous halo, respectively. FT‐IR spectra were recorded on a Bruker Alpha‐P FTIR spectrometer using attenuated total reflectance (ATR) equipment. Solvent extraction experiment was performed by placing a small piece (≈20 mg) of U‐PCL films into a 10 mL vial filled with THF. After the dissolution process in an oven at 25 °C for 24 h, the film was collected and dried at 60 °C under vacuum for 12 h. Gel fraction (*f*_g_) was calculated as

(3) $$f_{g}=W_{d}/W_{i}\times 100\%$$

where *W*_i_ (initial weight) and *W*_d_ (dried weight) are the weights of dried films before and after the THF solvent extraction. The self‐healing study was carried out by observation of scratches using an optical microscope (OM, Nikon 50iPol, Nikon) with a temperature controller (Instec mK1000, Instec, Boulder). The samples with a thickness of 0.5 mm were casted on the slide glass and scratched by a utility knife. The mechanical property analysis of U‐PCL vitrimers was carried out using a UTM (Instron LR5K, Lloyd Instruments). Rectangular shaped tensile bars (≈60 mm (*L*) × 5 mm (*W*) × 0.3 mm (*T*), gauge length = 20 mm) were stamped out from the films using a cutting die, and the tensile properties were measured at a strain rate of 0.0083 s^−1^ (10 mm min^−1^). At least three different samples were prepared from each film and tested in the UTM. All the tensile tests were performed at 25 ± 1 °C. For the lap‐shear test, two vitrimer blocks (≈30 mm (*L*) × 5 mm (*W*) × 0.3 mm (*T*), gauge length = 20 mm) were overlapped by the area of 7 × 5 mm^2^ and pressed under 10% compression within two Teflon sheets, followed by thermal treatment in a vacuum oven at 160 °C for 2 h. The scratch depth on the U‐PCL vitrimer films was observed using an alpha‐step surface profiler (*α*‐step DC50, KLA‐Tencor). Examination on flow properties of U‐PCL vitrimer was carried out using a Tinius Olsen MP1200 melt flow indexer (MFI). After preheating to the designated temperatures for 300 s, a standard load of 5.0 kg was applied and the flow of U‐PCL vitrimer was quantified. The MFI values were obtained four times and averaged out at each temperature. Rheological experiments were conducted on a strain‐controlled rheometer (ARES, TA Instruments) using a 25 mm parallel plate under a nitrogen environment. Frequency sweeps from 0.1 to 300 rad s^−1^ at 200 °C and 1% strain were performed on disk‐shaped specimen with a dimension of 25 mm diameter and 1 mm thickness.

## Conflict of Interest

The authors declare no conflict of interest.

## Supporting information

Supporting Information

Supplemental Movie 1

Supplemental Movie 2

Supplemental Movie 3

Supplemental Movie 4

## Data Availability

Research data are not shared.
