# Supplementary material for: A 4D Printable Shape Memory Vitrimer with Repairability and Recyclability through Network Architecture Tailoring from Commercial Poly(ε‐caprolactone)
Source: Adv Sci (Weinh). 2021 Oct 29;8(24):2103682. doi: 10.1002/advs.202103682 (PMC8693056; doi:10.1002/advs.202103682)
Supplement: Supplementary file 1 — Supporting Information [file ADVS-8-2103682-s001.pdf]

ADVANCED SCIENCE
Open Access

Supporting Information

for *Adv. Sci.*, DOI: 10.1002/advs.202103682

A 4D Printable Shape Memory Vitrimer with Repairability and Recyclability through Network Architecture Tailoring from Commercial Poly(ε-caprolactone)

*Jungho Joe, Jeehae Shin, Yong-Seok Choi, Jae Hyuk Hwang, Sang Hwa Kim, Jiseok Han, Bumsoo Park, Woohwa Lee, Sungmin Park,*Yong Seok Kim,* Dong-Gyun Kim**

Supporting Information

A 4D Printable Shape Memory Vitrimer with Repairability and Recyclability through Network Architecture Tailoring from Commercial Poly(ε-caprolactone)

Jungho Joe,‡ [a] Jeehae Shin,‡ [a] Yong-Seok Choi,[b] Jae Hyuk Hwang,[ac] Sang Hwa Kim,[ad] Jiseok Han,[ae] Bumsoo Park,[a] Woohwa Lee,[a] Sungmin Park,[a] Yong Seok Kim,*[af] Dong-Gyun Kim*[af]*

[a] Advanced Materials Division, Korea Research Institute of Chemical Technology, 141 Gajeong-ro, Yuseong-gu, Daejeon 34114, Republic of Korea.

[b] Composite Materials Application Research Center, Korea Institute of Science and Technology, 92 Chudong-ro, Bongdong-eup, Wanju-gun, Jeonbuk 55324, Republic of Korea.

[c] School of Chemical and Biological Engineering and Institute of Chemical Processes, Seoul National University, 599 Gwanak-ro, Gwanak-gu, Seoul 08826, Republic of Korea.

[d] Department of Chemical Engineering and Applied Chemistry, Chungnam National University, 99 Daehak-ro, Yuseong-gu, Daejeon 34134, Republic of Korea

[e] Department of Chemical Engineering and Applied Chemistry, Chungnam National University, 99 Daehak-ro, Yuseong-gu, Daejeon 34134, Republic of Korea

[f] Advanced Materials and Chemical Engineering, KRICT School, University of Science and Technology, 217 Gajeong-ro, Yuseong-gu, Daejeon 34114, Republic of Korea.

**Corresponding author: D.-G. Kim (E–mail: dgkim@krict.re.kr)*
S. Park (E–mail: parks@krict.re.kr)
Y. S. Kim (E–mail: yongskim@krict.re.kr)

†These authors contributed equally to this work.

Table of Contents

Synthesis and characterization of U-PCL vitrimer 3
Self-healing, welding, and reprocessing of U-PCL vitrimer 10
Flow behavior of U-PCL vitrimer 13
Dimensional stability of commercial PCL and U-PCL vitrimer at elevated temperatures 14
Reference for supporting information 14

Other Supplementary Material for this manuscript includes the following:

Movie S1. Shape memory behavior of U-PCL vitrimer film
Movie S2. Printing by a 3D pen
Movie S3. Dimensional stability of commercial PCL and U-PCL vitrimer at 80 ^{o}C (x10 faster)
Movie S4. Shape memory behavior of U-PCL vitrimer-based 3D-printed drawbridge

Synthesis and chraracterization of U-PCL vitrimer

Table S1. Chemical compositions of prepared U_a-PCL networks.

$\frac{[NCO_{PHMDI}]}{[OH_{PCL\ diol}]}$, a	PCL		PHMDI		$Zn(acac)_2$	
	(g)	(mmol)	(g)	(mmol)	(g)	(mmol)
1.0	2.500	1.250	0.471	0.865	0.118	0.448
1.2	2.500	1.250	0.526	0.966	0.118	0.448
1.3	2.500	1.250	0.581	1.067	0.118	0.448
1.5	2.500	1.250	0.686	1.260	0.118	0.448
1.7	2.500	1.250	0.791	1.453	0.118	0.448
2.0	2.500	1.250	0.897	1.648	0.118	0.448
2.3	2.500	1.250	1.050	1.929	0.118	0.448
2.9	2.500	1.250	1.320	2.425	0.118	0.448

Table S2. Physical properties of synthesized U_a-PCL networks.

$\frac{[NCO_{PHMDI}]}{[OH_{PCL\ diol}]}$, a	T_m ^{a} (°C)	T_c ^{a} (°C)	ΔH_m ^{b} (J g^{-1})	χ_c ^{b} (%)
1.0	48	17	53	39
1.2	46	11	51	38
1.3	46	10	49	36
1.5	46	2	32	24
1.7	41	-20	31	23

^{a} The melting temperature (T_m) and crystallization temperature (T_c) were obtained from the peak values of the melting endotherms and crystallization exotherms (in DSC), respectively. ^{b} The degree of crystallinity (χ_c) was determined by dividing the obtained melting enthalpy (ΔH_m) by the melting enthalpy for 100% crystalline PCL (ΔH°_m = 135 J g^{-1}).[S1]

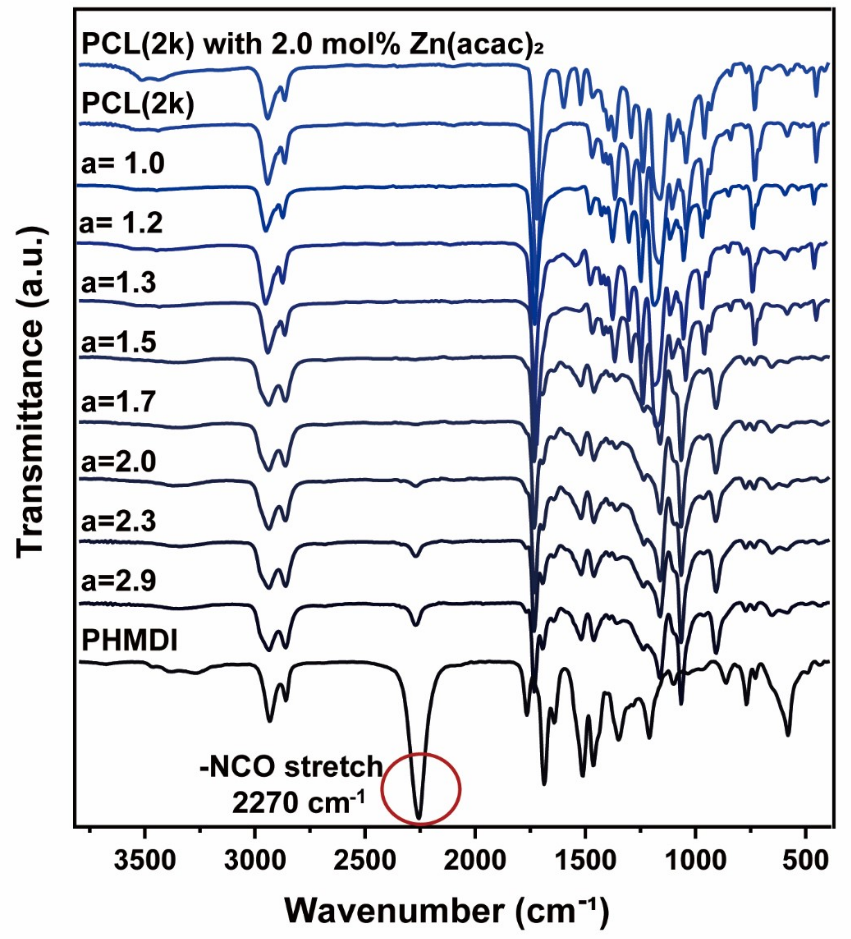

Figure S1. FT-IR spectra of the prepared set of U_a-PCL networks.

,

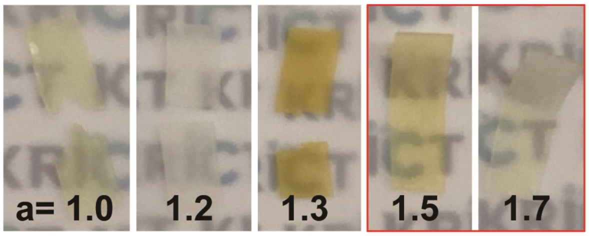

Figure S2. U_a-PCL network films upon folding. Undamaged samples are boxed in red.

Table S3. Chemical compositions of prepared $U_{1.7}$-PCL-PSA_b vitrimers.

OH_{PSA} (mol%), b	PCL		PHMDI		$Zn(acac)_2$		PSA	
	(g)	(mmol)	(g)	(mmol)	(g)	(mmol)	(g)	(mmol)
2	2.500	1.250	0.791	1.453	0.118	0.448	0.083	0.069
4	2.500	1.250	0.791	1.453	0.118	0.448	0.167	0.139
6	2.500	1.250	0.791	1.453	0.118	0.448	0.260	0.217
9	2.500	1.250	0.791	1.453	0.118	0.448	0.417	0.348
13	2.500	1.250	0.791	1.453	0.118	0.448	0.595	0.496
16	2.500	1.250	0.791	1.453	0.118	0.448	0.757	0.631

Table S4. Physical properties of synthesized $U_{1.7}$-PCL-PSA_b vitrimers.

OH_{PSA} (mol%), b	T_m ^{a} (°C)	T_c ^{a} (°C)	ΔH_m ^{b} (J g^{-1})	χ_c ^{b} (%)	f_g ^{c} (%)
2	48	8	33	24	49
4	41	-9	27	20	74
6	38	-10	20	20	87
9	31	-	7	5	83
13	-	-	-	-	89
16	-	-	-	-	90

^{a} The melting temperature (T_m) and crystallization temperature (T_c) were obtained from the peak values of the melting endotherms and crystallization exotherms (in DSC), respectively. ^{b} The degree of crystallinity (χ_c) was determined by dividing the obtained melting enthalpy (ΔH_m) by the melting enthalpy for 100% crystalline PCL (ΔH°_m = 135 J g^{-1}).[S1] ^{c} Gel fraction, obtained by $f_g = W_d / W_i \times 100\%$, where W_i (initial weight) and W_d (dried weight) are the weights of dried film before and after the THF solvent extraction.

Table S5. Chemical compositions of prepared $U_{1.7}$-PCL-PSA_6-Zn_c vitrimers.

Zn^{2+} (mol%), c	PCL		PHMDI		Zn(acac)2		PSA	
	(g)	(mmol)	(g)	(mmol)	(g)	(mmol)	(g)	(mmol)
12	2.500	1.250	0.791	1.453	0.749	2.841	0.260	0.217
8	2.500	1.250	0.791	1.453	0.477	1.809	0.260	0.217
6	2.500	1.250	0.791	1.453	0.350	1.328	0.260	0.217
4	2.500	1.250	0.791	1.453	0.229	0.869	0.260	0.217
2	2.500	1.250	0.791	1.453	0.118	0.448	0.260	0.217
1	2.500	1.250	0.791	1.453	0.056	0.211	0.260	0.217

Table S6. Physical properties of synthesized $U_{1.7}$-PCL-PSA_6-Zn_c vitrimers.

Zn^{2+} (mol%), c	T_m ^{a} (°C)	T_c ^{a} (°C)	ΔH_m ^{b} (J g^{-1})	χ_c ^{b} (%)	f_g ^{c}(%)
12	35	-	3	2	36
8	36	-	3	3	46
6	36	-	10	13	62
4	38	-19	18	18	75
2	38	-10	20	20	87
1	38	-12	25	25	90

^{a} The melting temperature (T_m) and crystallization temperature (T_c) were obtained from the peak values of the melting endotherms and crystallization exotherms (in DSC), respectively. ^{b} The degree of crystallinity (χ_c) was determined by dividing the obtained melting enthalpy (ΔH_m) by the melting enthalpy for 100% crystalline PCL (ΔH°_m = 135 J g^{-1}).[S1] ^{c} Gel fraction, obtained by $f_g = W_d / W_i \times 100\%$, where W_i (initial weight) and W_d (dried weight) are the weights of dried film before and after the THF solvent extraction.

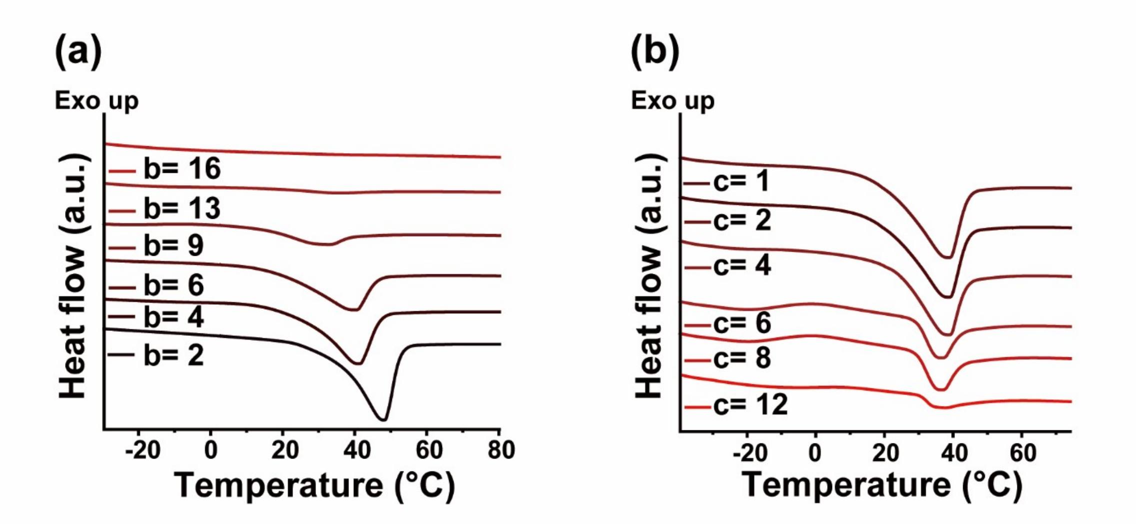

Figure S3. DSC thermograms of a) $U_{1.7}$-PCL-PSA_b and b) $U_{1.7}$-PCL-PSA_6-Zn_c vitrimers during the second heating scan at a rate of 10°C min^{-1} from –80°C to 200°C.

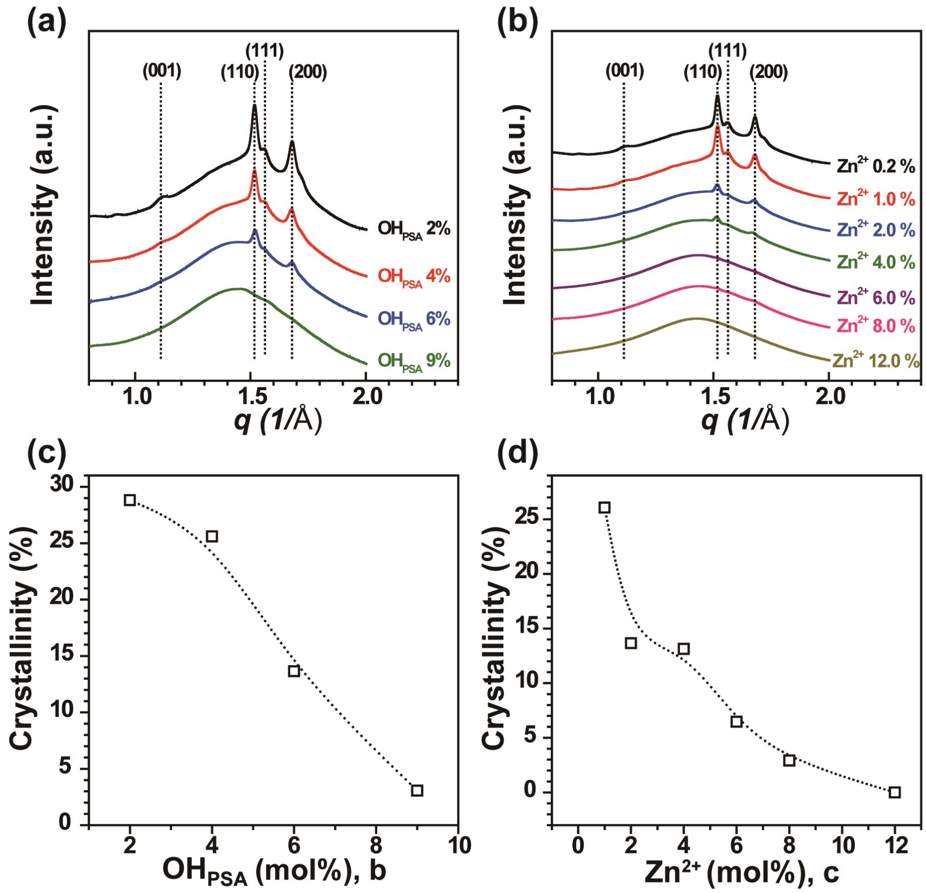

Figure S4. WAXD analysis of the effect of PSA and $Zn(acac)_2$ contents in $U_{1.7}$-PCL vitrimers. 1D intensity profiles for a) U-$PCL_{1.7}$-PSA_b-Zn_4 vitrimers depending on PSA content and b) U-$PCL_{1.7}$-PSA_6-Zn_c vitrimers depending on $Zn(acac)_2$ content. Crystallinty of c) U-$PCL_{1.7}$-PSA_b-Zn_4 vitrimers as a function of PSA content and d) U-$PCL_{1.7}$-PSA_6-Zn_c vitrimers as a function of $Zn(acac)_2$ content, determined from WAXD data.

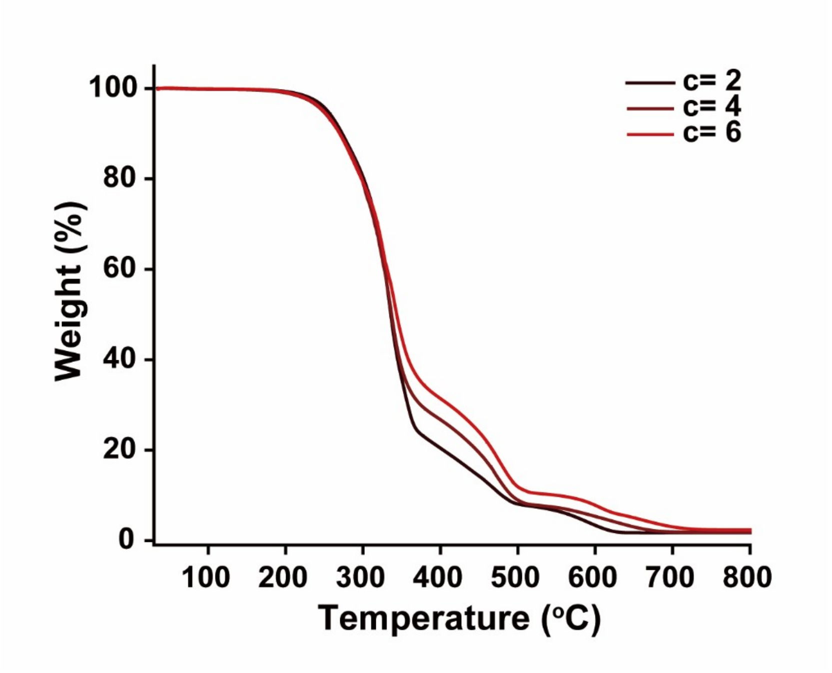

Figure S5. TGA curves of $U_{1.7}$-PCL-PSA_6-Zn_c vitrimers.

Self-healing, welding, and reprocessing of U-PCL vitrimer

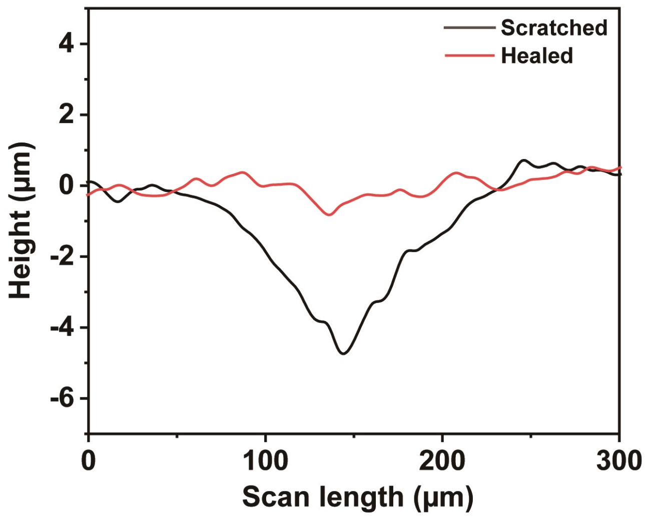

Figure S6. Depth profiles of a scratched and healed $U_{1.7}$-PCL-PSA_6-Zn_4 vitrimer film.

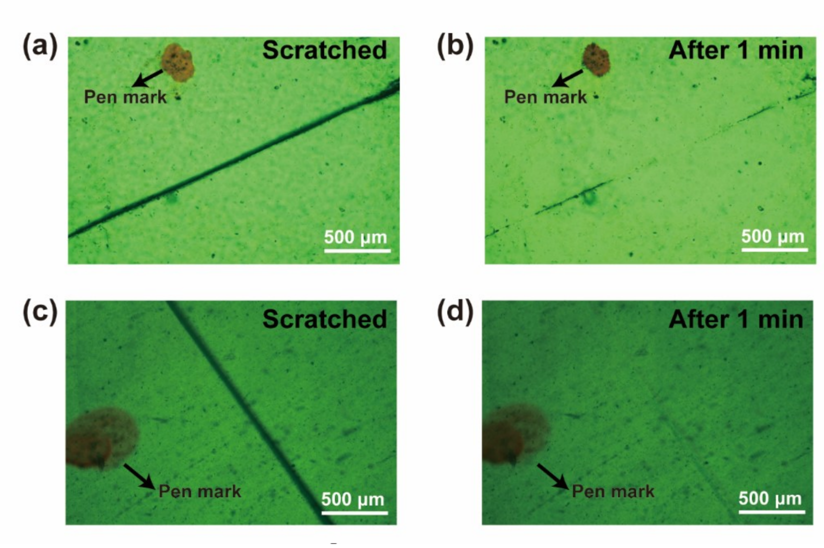

Figure S7. Optical microscopy images of scratched $U_{1.7}$-PCL-PSA_6-Zn_c after being self-healed for 0, 1 min at 160 °C. a-b) $U_{1.7}$-PCL-PSA_6-Zn_2; c-d) $U_{1.7}$-PCL-PSA_6-Zn_4. Left panel corresponds 0 min and right panel corresponds 1 min at 160 °C.

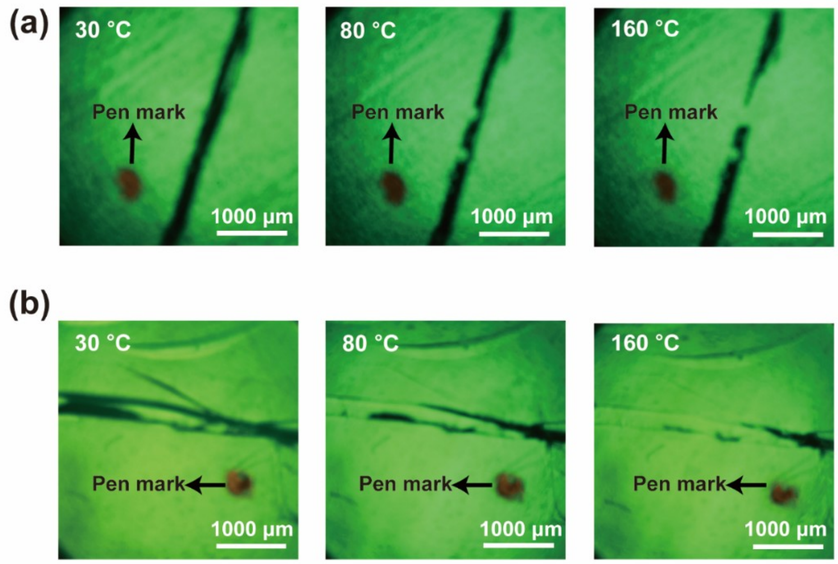

Figure S8. Optical microscopy images of scratched a) $U_{1.7}$-PCL-PSA_6-Zn_2 and b) $U_{1.7}$-PCL-PSA_6-Zn_4 after being self-repaired at a heating rate of 10°C min^{-1} from 30°C to 160°C.

Table S7. Mechanical properties of pristine and welded $U_{1.7}$-PCL-PSA$_6$-Zn$_4$ vitrimer films.

	E^a (MPa)	$\sigma_u{}^a$ (MPa)	$\varepsilon_b{}^a$ (%)	$U_T{}^a$ (MJ m^{-3})
Pristine	59.1 ± 14	2.66 ± 0.2	26.6 ± 2	53.3 ± 6
Welded	58.9 ± 1	1.58 ± 0.1	14.9 ± 1	24.2 ± 3

[a] Determined from tensile testing at 25±1 °C and a strain rate of 0.0083 s^{-1}, where E, σ_u, ε_b, and U_T are elastic modulus, ultimate tensile stress, strain at break, and toughness, respectively. Welded under ≈10% compression at 160 °C for 2 h.

Table S8. Mechanical properties of $U_{1.7}$-PCL-PSA$_6$-Zn$_4$ vitrimer films with different process numbers.

Process number	E^a (MPa)	$\sigma_u{}^a$ (MPa)	$\varepsilon_b{}^a$ (%)	$U_T{}^a$ (MJ m^{-3})
1 (pristine)	59.1 ± 14	2.66 ± 0.2	26.6 ± 3	53.3 ± 6
2	63.3 ± 15	2.36 ± 0.2	15.7 ± 3	26.7 ± 8
3	48.0 ± 3	2.11 ± 0.2	15.5 ± 3	27.4 ± 5
4	56.6 ± 8	2.13 ± 0.2	15.9 ± 3	24.5 ± 3

[a] Determined from tensile testing at 25±1 °C and a strain rate of 0.0083 s^{-1}, where E, σ_u, ε_b, and U_T are elastic modulus, ultimate tensile stress, strain at break, and toughness, respectively. Reprocessed under 10 MPa at 170 °C for 0.5 h.

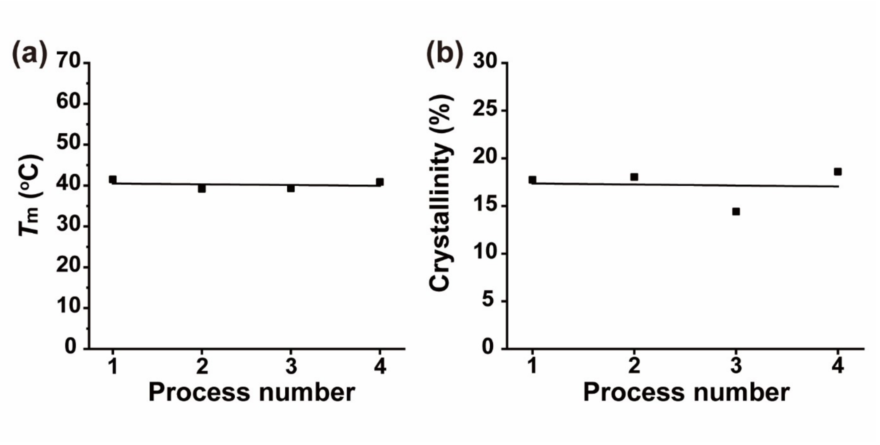

Figure S9. a) Melting temperatures and b) crystallinity of reprocessed $U_{1.7}$-PCL-PSA$_6$-Zn$_4$ vitrimer films.

Flow behavior of U-PCL vitrimer

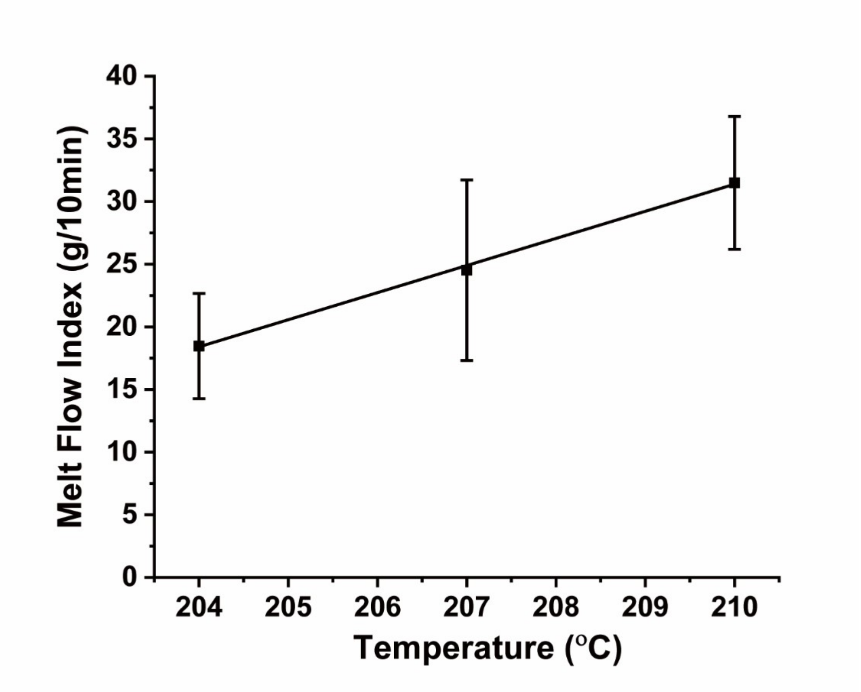

Figure S10. Melt-flow index of $U_{1.7}$-PCL-PSA_6-Zn_4 vitrimer conducted at 204, 207, and 210 °C. (Test load= 5.0 kg, pre-heat= 300 s).

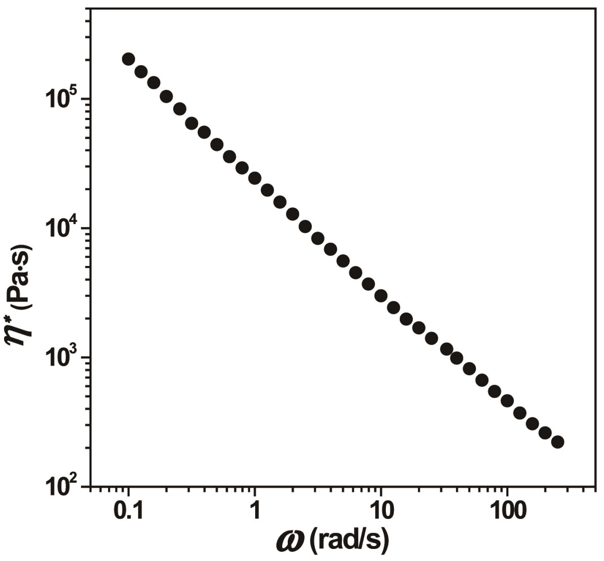

Figure S11. Complex viscosity as a function of angular frequency for $U_{1.7}$-PCL-PSA_6-Zn_4 vitrimer at 200 °C.

Dimensional stability of commercial PCL and U-PCL vitrimer at elevated temperatures

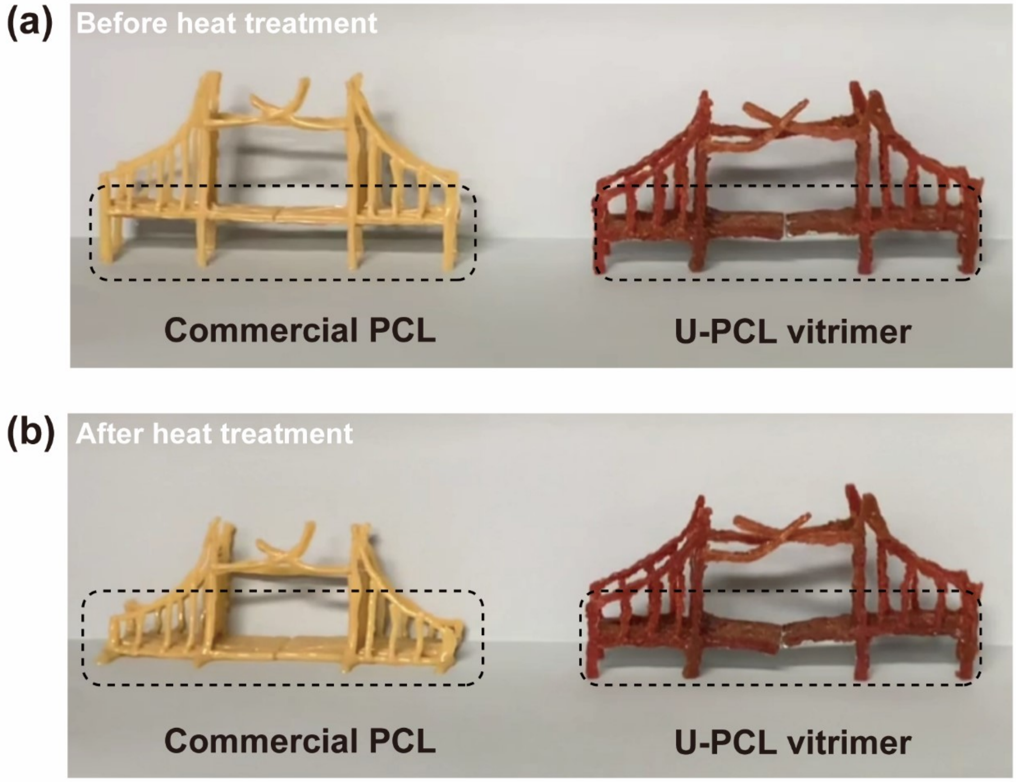

Figure S12. Comparison of dimensional stability between U-PCL vitrimer printed bridge and commercially available PCL printed bridge in an oven at 80°C for 5 minutes.

Reference for supporting information

[S1] Nagata, M.; Yamamoto, Y. *J. Polym. Sci., Part A: Polym. Chem.* **2009**, *47*, 2422.
